# Expression Profiling of Mitochondrial Voltage-Dependent Anion Channel-1 Associated Genes Predicts Recurrence-Free Survival in Human Carcinomas

**DOI:** 10.1371/journal.pone.0110094

**Published:** 2014-10-15

**Authors:** Jae-Hong Ko, Wanjun Gu, Inja Lim, Tong Zhou, Hyoweon Bang

**Affiliations:** 1 Department of Physiology, College of Medicine, Chung-Ang University, Seoul, South Korea; 2 Research Center for Learning Sciences, Southeast University, Nanjing, Jiangsu, China; 3 Department of Medicine, University of Arizona, Tucson, Arizona, United States of America; University College London, United Kingdom

## Abstract

**Background:**

Mitochondrial voltage-dependent anion channels (VDACs) play a key role in mitochondria-mediated apoptosis. Both *in vivo* and *in vitro* evidences indicate that VDACs are actively involved in tumor progression. Specifically, VDAC-1, one member of the VDAC family, was thought to be a potential anti-cancer therapeutic target. Our previous study demonstrated that the human gene *VDAC1* (encoding the VDAC-1 isoform) was significantly up-regulated in lung tumor tissue compared with normal tissue. Also, we found a significant positive correlation between the gene expression of *VDAC1* and histological grade in breast cancer. However, the prognostic power of *VDAC1* and its associated genes in human cancers is largely unknown.

**Methods:**

We systematically analyzed the expression pattern of *VDAC1* and its interacting genes in breast, colon, liver, lung, pancreatic, and thyroid cancers. The genes differentially expressed between normal and tumor tissues in human carcinomas were identified.

**Results:**

The expression level of *VDAC1* was uniformly up-regulated in tumor tissue compared with normal tissue in breast, colon, liver, lung, pancreatic, and thyroid cancers. Forty-four *VDAC1* interacting genes were identified as being commonly differentially expressed between normal and tumor tissues in human carcinomas. We designated *VDAC1* and the 44 dysregulated interacting genes as the *VDAC1* associated gene signature (VAG). We demonstrate that the VAG signature is a robust prognostic biomarker to predict recurrence-free survival in breast, colon, and lung cancers, and is independent of standard clinical and pathological prognostic factors.

**Conclusions:**

VAG represents a promising prognostic biomarker in human cancers, which may enhance prediction accuracy in identifying patients at higher risk for recurrence. Future therapies aimed specifically at *VDAC1* associated genes may lead to novel agents in the treatment of cancer.

## Background

Mitochondrial voltage-dependent anion channels (VDACs) are a class of porin ion channels located on the outer membrane of mitochondria [1]. VDACs allow diffusion of small hydrophilic molecules [2], [3], which play an important role in regulating metabolic and energetic flux across the outer mitochondrial membrane by transporting ions and molecules such as ATP, ADP, pyruvate, malate, and other metabolites [4]. Also, VDACs are known to form channels through the plasma membrane [5], which are involved in cell volume regulation [5], [6]. In addition, VDACs have been reported to play a key role in mitochondria-mediated apoptosis [7], [8], [9]. VDACs are the major permeability pathways through the outer mitochondrial membrane. During apoptosis, increased permeability of VDACs allows for the release of apoptogenic proteins to the cytosol, which is strongly associated with cell death [10].

Given their key role in apoptosis, VDACs are being studied as cancer-specific targets [11]. Both *in vivo* and *in vitro* evidences indicate that VDACs are actively involved in tumor progression [7], [11], [12], [13]. Specifically, voltage dependent anion channel-1 (VDAC-1), one member of the VDAC family, is thought to be a potential anti-cancer therapeutic target [13], [14], [15]. Currently, there are three isoforms of VDACs that have been characterized in mammalian tissues, including VDAC-1, voltage dependent anion channel-2 (VDAC-2), and voltage dependent anion channel-3 (VDAC-3). VDAC-1 is the most abundant in comparison to VDAC-2 and VDAC-3. More importantly, VDAC-1 is ubiquitously expressed across all other tissues types [14]. The complex of VDAC-1 and glycolytic enzyme hexokinase regulates metabolite trafficking through the outer membrane channels and provides cancer cells with metabolic advantages [7], [16], which protects against mitochondria-mediated apoptosis. Knockdown of VDAC-1 can lead to a block in cancer cell proliferation in nude mice subcutaneously injected with HeLa cancer cells [15]. Overall, VDAC-1 is the most physiologically and metabolically important isoform among the VDAC family.

In our previous study, we demonstrated that the human gene of the VDAC-1 isoform (*VDAC1*) is significantly up-regulated in lung tumor tissue compared with normal tissue [17]. Also, we found a significant positive correlation between the gene expression of *VDAC1* and histological grade in breast cancer [18], which suggests that the expression pattern of *VDAC1* and its interacting genes may serve as indicators for cancer prognosis. In fact, gene expression profiling of clinical tumors has led to the discovery of numerous molecular biomarkers for prognosis [19]. For example, meta-analysis on gene expression by Grills *et al.* suggested *VDAC1* as a potential predictor of poor outcome in early stage non-small cell lung cancer [20]. However, the prognostic power of *VDAC1* and its associated genes in human cancers is still largely unknown.

In this study, we systematically analyzed the expression pattern of *VDAC1* associated genes in cancers. We looked to identify a molecular signature consisting of multiple genes including *VDAC1* and its interacting genes that are implicated in the pathology of human carcinomas. We first compared the expression of *VDAC1* associated genes between normal and tumor tissues in six different carcinomas. Forty-four genes were identified as being commonly differentially expressed between normal and tumor tissues. Next, we investigated the prognostic power of the *VDAC1* associated genes in breast, colon, and lung cancers. We demonstrate that the *VDAC1* associated gene signature is a robust prognostic biomarker to predict recurrence-free survival in breast, colon, and lung cancers, and is independent of standard clinical and pathological prognostic factors. Notably, we derived a novel gene signature associated with mitochondria-mediated apoptosis that predicts clinical outcome in human cancers.

## Methods

### Gene expression data of paired normal and tumor tissues

We collected gene expression data of paired normal and tumor tissues for breast (GSE15852) [21], colon (GSE23878) [22], liver (GSE14520) [23], lung (GSE18842) [24], pancreatic (GSE15471) [25], and thyroid (GSE33630) cancers from the Gene Expression Omnibus (GEO) database. We used these datasets to identify differentially expressed genes between normal and tumor tissues for each type of cancer. The platform information for each dataset is listed in Table S1.

### Gene expression data for cancer survival analysis

Training and validation cohorts were constructed for breast, colon, and lung cancers. From the GEO database, we collected the expression datasets with available information on recurrence-free survival for breast (GSE21653 [26] for training and GSE25066 [27] for validation), colon (GSE17536 [28] for training and GSE39582 [29] for validation), and lung (GSE8894 [30] for training and GSE31210 [31] for validation) cancers (Table S1). These datasets were chosen based on the large number of samples and the availability of clinical outcome data.

### Microarray data preprocessing

The GC robust multichip average algorithm [32] was used to summarize the expression level of each probe set for the microarray data of paired normal and tumor tissues. Only the probe sets present (determined by function “mas5calls” in the Bioconductor “affy” package) in at least two thirds of the samples were retained. We further limited our analysis to the probe sets with unique annotations and removed genes on chromosomes X and Y to avoid potential confounding factors. For the gene with multiple probe sets, we use the average expression value of all probe sets that map to the gene. The preprocessed training and validation datasets are presented in Data S1.

### Identification of genes interacting with VDAC1

Information on genes interacting with *VDAC1* was obtained from GeneCards [33], [34] and BioGRID [35]. We also included several genes known to interact with *VDAC1*, including *HK2*, *MMP2*, and *MMP9* [36]. In total, we collected 342 well-annotated genes which interact with *VDAC1* (Table S2), according to the definition provided by BioGRID [35], UniProtKB [37], [38], MINT [39], [40], I2D [41], [42], STRING [43], [44], and published literature [36]. We also inferred the expression-based regulatory network of these interacting genes by GENIE3 [45], which is a gene regulatory network inference algorithm based on variable selection with ensembles of regression trees. The R script provided by the authors of GENIE3 was applied in this study.

### Patient risk score

For each cancer training dataset, univariate Cox proportional hazards regression was used to evaluate the association between recurrence-free survival and expression of commonly differentially expressed genes between normal and tumor tissues across breast, colon, liver, lung, pancreatic, and thyroid cancers. A risk score was then calculated for each patient using a linear combination of gene expression weighted by the Wald statistic (ratio of regression coefficient to its standard error) as shown below:
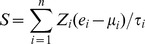
Here, *S* is the risk score of the patient; *n* is the number of differentially expressed genes; *Z_i_* denotes the Wald statistic of gene *i*; *e_i_* denotes the expression level of gene *i*; and *μ_i_* and *τ_i_* are the mean and standard deviation of the gene expression values for gene *i* across all samples, respectively. Patients were then divided into high- and low-score groups with the median of the risk score as the threshold value. A higher risk score implies a poor outcome. The scoring system and the associated scaling coefficients were fixed based on the training cohorts and then evaluated in the validation cohorts. All the statistical analyses were conducted by the R platform.

## Results

### Expression of VDAC1 and its interacting genes in human carcinomas

**Figure 1:**
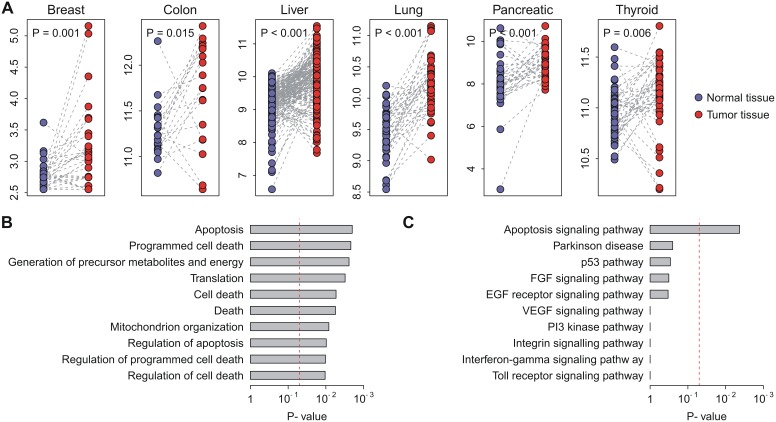
*VDAC1* and its interacting genes. (A) *VDAC1* is up-regulated in tumor tissues in breast, colon, liver, lung, pancreatic, and thyroid cancers. Paired normal and tumor tissues were included in the comparison. Y-axis: log_2_-transformed expression values. (B) The top 10 GO biological process terms associated with dysregulated *VDAC1* interacting genes. Forty-four *VDAC1* interacting genes were identified as being commonly differentially expressed between normal and tumor tissues in human carcinomas. The *P*-values were calculated by Fisher’s exact test. The red dash line denotes the significance level of 0.05. (C) The top ten PANTHER pathway terms associated with dysregulated *VDAC1* interacting genes. The *P*-values were calculated by Fisher’s exact test. The red dash line denotes the significance level of 0.05.

We first explored the difference in expression level of *VDAC1* between normal and tumor tissues in several human carcinomas. Paired normal and tumor tissues from 43 breast, 19 colon, 214 liver, 44 lung, 36 pancreatic, and 44 thyroid cancer patients were included. Paired t-tests indicated that *VDAC1* was uniformly up-regulated in tumor tissues across all six cancer types, with a fold change of 1.25 in breast, 1.32 in colon, 1.64 in liver, 1.67 in lung, 1.91 in pancreatic, and 1.14 in thyroid cancers (Figure 1A).

We next checked the difference in expression levels of genes interacting with *VDAC1* between normal and tumor tissues. We collected 342 *VDAC1* interacting genes, by which the proteins encoded have been reported to interact with VDAC-1 (Table S2). Paired t-test was used to detect the differentially expressed genes between normal and tumor tissues. In total, 44 *VDAC1* interacting genes were identified as being differentially expressed in the accordant direction between the two groups (*P*<0.05 after Benjamini-Hochberg adjustment) in at least four cancer types (Table S3). As expected, these genes were significantly enriched by Gene Ontology (GO) biological process terms [46] involved in apoptosis, such as “Apoptosis”, “Programmed cell death”, and “Generation of precursor metabolites and energy” (Figure 1B). Also, pathway analysis in the PANTHER database [47] confirmed that the top term associated with the 44 *VDAC1* interacting genes is “Apoptosis signaling pathway” (Figure 1C). We designated *VDAC1* and the 44 dysregulated interacting genes as the *VDAC1* associated gene signature (VAG) (Table 1).

**Table 1:** *VDAC1* associated gene signature (VAG).

Gene symbol	Gene title
*VDAC1*	voltage-dependent anion channel 1
*ACADVL*	acyl-CoA dehydrogenase, very long chain
*AGK*	acylglycerol kinase
*AP2M1*	adaptor-related protein complex 2, mu 1 subunit
*ATP6V1A*	ATPase, H+ transporting, lysosomal 70 kDa, V1 subunit A
*BCL2L1*	BCL2-like 1
*CDK2*	cyclin-dependent kinase 2
*COX4I1*	cytochrome c oxidase subunit IV isoform 1
*CYCS*	cytochrome c, somatic
*DAP3*	death associated protein 3
*DBT*	dihydrolipoamide branched chain transacylase E2
*DENR*	density-regulated protein
*DHX30*	DEAH (Asp-Glu-Ala-His) box polypeptide 30
*ECI1*	enoyl-CoA delta isomerase 1
*EIF6*	eukaryotic translation initiation factor 6
*FLAD1*	Flavin Adenine Dinucleotide Synthetase 1
*GAPDH*	glyceraldehyde-3-phosphate dehydrogenase
*GSN*	gelsolin
*GSTK1*	glutathione S-transferase kappa 1
*HADHA*	hydroxyacyl-CoA dehydrogenase/3-ketoacyl-CoA thiolase/enoyl-CoA hydratase (trifunctional protein), alpha subunit
*HAUS3*	HAUS augmin-like complex, subunit 3
*IGF2BP2*	insulin-like growth factor 2 mRNA binding protein 2
*KIAA0391*	KIAA0391
*KIF5B*	kinesin family member 5B
*LONP1*	lon peptidase 1, mitochondrial
*MAPK1*	mitogen-activated protein kinase 1
*MCAT*	malonyl CoA:ACP acyltransferase (mitochondrial)
*MCL1*	myeloid cell leukemia sequence 1 (BCL2-related)
*MDC1*	mediator of DNA-damage checkpoint 1
*MRPL3*	mitochondrial ribosomal protein L3
*MRPL9*	mitochondrial ribosomal protein L9
*MRPS10*	mitochondrial ribosomal protein S10
*MRPS17*	mitochondrial ribosomal protein S17
*MTERFD1*	MTERF domain containing 1
*MTPAP*	Mitochondrial poly(A) polymerase
*MUT*	methylmalonyl CoA mutase
*PANK2*	pantothenate kinase 2
*PPID*	Peptidylprolyl isomerase D
*RNGTT*	RNA guanylyltransferase and 5′-phosphatase
*TFB1M*	transcription factor B1, mitochondrial
*TIAL1*	TIA1 cytotoxic granule-associated RNA binding protein-like 1
*TMX1*	thioredoxin-related transmembrane protein 1
*TOMM20*	translocase of outer mitochondrial membrane 20 homolog (yeast)
*TUBA4A*	tubulin, alpha 4a
*YWHAB*	tyrosine 3-monooxygenase/tryptophan 5-monooxygenase activation protein, beta polypeptide

To investigate the inherent relationship in gene expression among *VDAC1* and its interacting genes, we computed the gene regulatory network for VAG using GENIE3 [45], a state-of-the-art per-gene network inference algorithm. The weighted adjacency matrix was computed for breast, colon, liver, lung, pancreatic, and thyroid cancers. The element *w_ij_* in the weighted adjacency matrix gives the importance of the link from regulatory gene *i* to target gene *j*. We used the sum of the weighted adjacency matrix to measure the inherent relationship in gene expression among *VDAC1* and its interacting genes. We assumed that the linkage in VAG expression is stronger than random pattern. Therefore, we performed a resampling test to check whether the association in expression among VAG was by chance or not. We generated 1,000 random gene signatures with identical size as VAG. The weighted adjacency matrix was computed for each resampled gene set using GENIE3. We found that the sum of the weighted adjacency matrix of VAG was significantly larger than that of the randomized signature (*P* = 0.003 for breast cancer and *P*<0.001 for colon, liver, lung, pancreatic, and thyroid cancers) (Figure S1), which confirmed the inherent relationship in gene expression among *VDAC1* and its interacting genes.

### VAG predicts recurrence-free survival in breast, colon, and lung cancers

We hypothesized that the apoptosis associated gene signature, VAG, is predictive of tumor outcome in cancer patients. We constructed a risk scoring system that combined gene expression of VAG with risk for recurrence in the training dataset (see Methods for details). VAG-positive patients were defined as those having risk score greater than the group median. As expected, there was a significantly reduced recurrence-free survival for VAG-positive patients in the training cohorts (Figure S2 and Table 2).

**Table 2:** Cox proportional hazards regression of survival by VAG status in breast, colon, and lung cancers.

	Training cohort			Validation cohort		
Cancer	Hazardratio	95% confidenceinterval	*P*-value	Hazardratio	95% confidenceinterval	*P*-value
Breast	1.75	(1.13, 2.71)	1.3×10^−2^	2.19	(1.48, 3.24)	8.0×10^−5^
Colon	5.70	(2.37, 13.71)	1.0×10^−4^	1.48	(1.10, 2.00)	9.4×10^−3^
Lung	1.78	(1.10, 2.88)	1.8×10^−2^	4.35	(2.40, 7.88)	1.3×10^−6^

**Figure 2:**
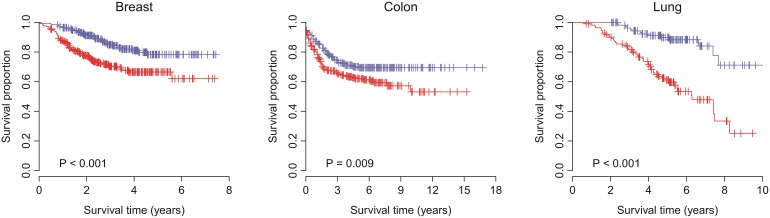
Kaplan-Meier curves for patients in the validation cohorts. The expression of VAG predicts poor recurrence-free survival in breast, colon, and lung cancers. Red curves are for VAG-positive patients while blue curves are for VAG-negative patients. VAG-positive patients were defined as those having a risk score greater than the group median. *P*-values were calculated by log-rank tests for the differences in survival between VAG-positive and -negative groups.

We next tested the ability of the VAG based risk score to classify patients into prognostic groups in the independent validation cohorts. Kaplan-Meier survival curves demonstrated a significantly reduced recurrence-free survival for VAG-positive patients in the validation cohorts for breast (*P*<0.001), colon (*P* = 0.009), and lung (*P*<0.001) cancers (Figure 2). Univariate Cox proportional hazards regression indicated that VAG-positive patients had an increased risk for recurrence of 2.19-fold in breast, 1.48-fold in colon, and 4.35-fold in lung cancers (Table 2). These findings collectively indicated that VAG is predictive of recurrence-free survival in multiple human cancers. However, we found that *VDAC1* expression itself is not predictive of recurrence-free survival in both training and validation datasests (Table S4).

### Non-random prognostic power of VAG

A computational study by Venet *et al.* pointed out that most random gene expression signatures are significantly associated with breast cancer outcome [48]. They compared 47 published breast cancer outcome signatures to signatures made of random genes. Roughly 60% of the published signature were not significantly better than random signatures of identical size.

**Figure 3:**
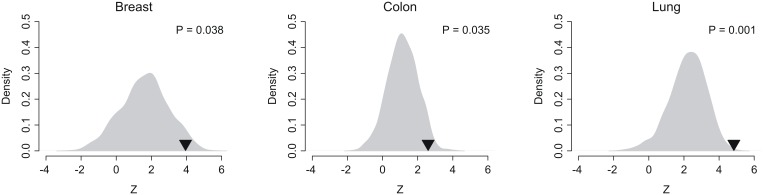
Non-random prognostic power of VAG in breast, colon, and lung cancers. *Z* denotes the Wald statistic, the ratio of Cox regression coefficient to its standard error. The black triangles stand for the *Z* values of VAG. The grey areas show the distribution of *Z* values for the 1,000 resampled gene signatures with identical size as VAG under the null hypothesis of no association between VAG and recurrence-free survival. One-tailed *P*-values for the right tail of the sampling distribution were calculated.

Here, we performed a resampling test to check whether the prognostic power of VAG was significantly better than random gene signatures. We constructed 1,000 random gene signatures with identical size as VAG. Cox proportional hazards regression of survival was conducted for each resampled gene signature. The association between each random gene signature and recurrence-free survival was measured by the Wald statistic, the ratio of Cox regression coefficient to its standard error. Our alternative hypothesis was that the Wald statistic of VAG should be more positive than expected by chance if the prognostic power of VAG was significantly better than random gene signatures. We found that, in the validation cohorts, we could reject the null hypothesis that the association between VAG and recurrence-free survival is by chance. The Wald statistic of VAG was significantly larger than that of randomized gene signatures (*P* = 0.038 for breast cancer, *P* = 0.035 for colon cancer, and *P* = 0.001 for lung cancer) (Figure 3).

Next, we compared the prognostic power of VAG with the other cancer-related genes. The cancer-related genes were identified as being differentially expressed in the accordant direction between normal and tumor tissues (*P*<0.05 after Benjamini-Hochberg adjustment) in at least four cancer types. In total, we collected 1,357 cancer-related genes that don’t interact with *VDAC1* (Table S5). We performed a resampling test to check whether the predictive power of VAG was statistically better than the other cancer-related genes. We randomly picked up 45 genes from the pool of cancer-related genes. The performance of the random gene set was quantified by the sum of the Wald statistic of the validation cohorts. By 1,000 times of randomization, we found that the prognostic power of VAG was significantly better than that of the random gene signatures that were composed of cancer-related genes (*P* = 0.014) (Figure S3).

### Multivariate analysis with clinical and pathological factors

**Figure 4:**
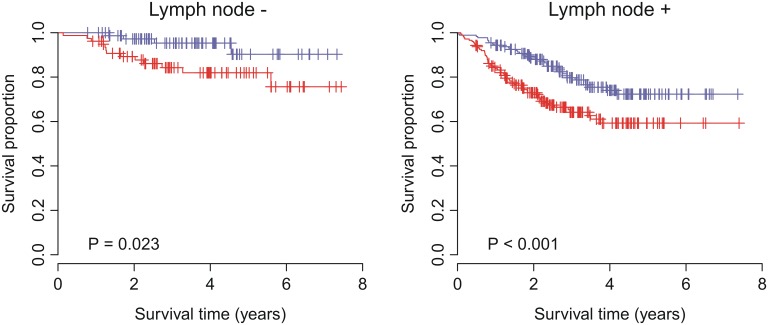
Kaplan-Meier curves for breast cancer patients from validation cohort grouped by lymph node status. Red curves are for VAG-positive patients while blue curves are for VAG-negative patients. VAG-positive patients were defined as those having a risk score greater than the group median. *P*-values were calculated by log-rank tests for the differences in survival between VAG-positive and -negative groups.

**Figure 5:**
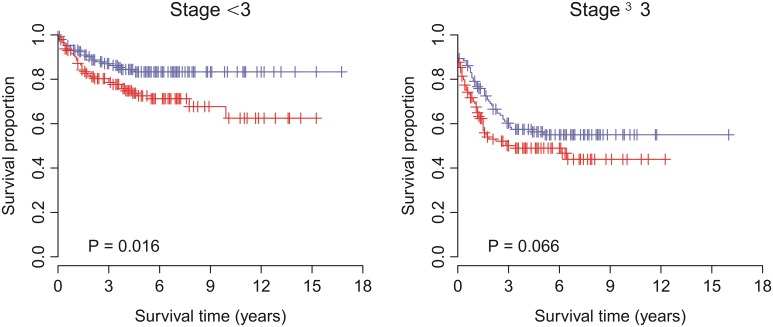
Kaplan-Meier curves for colon cancer patients from the validation cohort stratified by stage. Red curves are for VAG-positive patients while blue curves are for VAG-negative patients. VAG-positive patients were defined as those having a risk score greater than the group median. *P*-values were calculated by log-rank tests for the differences in survival between VAG-positive and -negative groups.

To investigate the performance of VAG in comparison with standard clinical and pathological factors associated with prognosis in human carcinomas, multivariate analyses were conducted for breast, colon, and lung cancers. For breast cancer, we considered factors including patient age, lymph node status, histological grade, tumor size, estrogen receptor (ER) status, and progesterone receptor (PR) status. For colon cancer, we took age, gender, stage, and *TP53*, *BRAF*, and *KRAS* mutation status into account. For lung cancer, factors, such as age, gender, stage, smoking history, Myc protein level, and *EGFR*/*KRAS*/*ALK* alteration status were included in the multivariate model. Multivariate Cox proportional hazards regression of survival indicated that, in the validation cohorts, VAG dichotomized status remained a significant covariate in relation to the clinical and pathological factors for breast (*P* = 0.030), colon (*P* = 0.018), and lung (*P*<0.001) cancers (Table 3). VAG was the most significant covariate in lung cancer. However, lymph node status and stage appeared to be the most significant covariate in breast and colon cancers, respectively. Accordingly, for breast cancer, we further stratified the patients by lymph node status. For breast cancer patients without or with lymph node involvement, VAG-positive patients had a 3.41-fold (*P* = 0.032) and 2.00-fold (*P* = 0.001) increased risk for recurrence, respectively (Figure 4). For colon cancer patients with stage <3 or ≥3, VAG-positive patients had a 1.89-fold (*P* = 0.018) and 1.40-fold (*P* = 0.069) increased risk for recurrence, respectively (Figure 5). Taken together, these results suggest that VAG enhances the identification of cancer patients at greater risk for recurrence.

**Table 3:** Multivariate Cox proportional hazards regression of survival in the validation cohorts.

Cancer	Covariate	Hazard ratio	95% confidence interval	*P*-value
Breast	VAG + vs. −	1.68	(1.05, 2.68)	3.0×10^−2^
	Age (per year)	1.00	(0.98, 1.02)	9.5×10^−1^
	Lymph node + vs. −	2.78	(1.60, 4.82)	3.0×10^−4^
	Grade 3 vs. 1,2	0.71	(0.43, 1.17)	1.8×10^−1^
	Tumor size ≥T3 vs. <T3	1.78	(1.19, 2.64)	4.5×10^−3^
	ER + vs. −	0.52	(0.30, 0.89)	1.8×10^−2^
	PR + vs. −	0.68	(0.41, 1.16)	1.6×10^−1^
Colon	VAG + vs. −	1.59	(1.08, 2.33)	1.8×10^−2^
	Age (per year)	1.00	(0.98, 1.01)	5.9×10^−1^
	Gender male vs. female	1.30	(0.88, 1.93)	1.9×10^−1^
	Stage III, IV vs. I, II	2.23	(1.49, 3.34)	9.4×10^−5^
	*TP53* mutation + vs. −	1.28	(0.87, 1.90)	2.1×10^−1^
	*BRAF* mutation + vs. −	1.34	(0.64, 2.80)	4.4×10^−1^
	*KRAS* mutation + vs. −	1.54	(1.03, 2.30)	3.5×10^−2^
Lung	VAG + vs. −	4.30	(2.28, 8.12)	6.5×10^−6^
	Age (per year)	1.05	(1.01, 1.09)	1.5×10^−2^
	Gender male vs. female	0.95	(0.47, 1.91)	8.8×10^−1^
	Smoking + vs. −	0.92	(0.45, 1.88)	8.2×10^−1^
	Stage II vs. I	2.65	(1.57, 4.47)	2.8×10^−4^
	*EGFR*/*KRAS*/*ALK* alteration + vs. −	0.49	(0.29, 0.83)	8.4×10^−3^
	Myc level high vs. low	0.72	(0.28, 1.84)	4.9×10^−1^

## Discussion

Mitochondria are membrane-bound organelles found in most eukaryotic cells, which have been recognized for their multiple functions in metabolism, energy transduction, ion transport, inheritance, signaling, and cell death [49]. The outer membrane of mitochondria encloses a periplasmic space where proteins associated with cell death are stored [50]. VDAC-1 is one of the major proteins located on the outer mitochondrial membrane, which may inhibit apoptosis and promote tumorigenesis through specific interactions with enzymes favoring glycolysis [50]. The complex of VDAC-1 and hexokinase regulates metabolite transportation through the outer membrane channels and provides cancer cells with metabolic advantages [11]. Therefore, any change in VDAC-1 expression may lead to alteration in cancer cell metabolism, ultimately promoting or inhibiting cell death [11].

Because VDAC-1 plays a key role in cancer cell fate through different signaling mechanisms, we investigated the expression pattern of *VDAC1* and its interacting genes. Firstly, we found that *VDAC1* is uniformly up-regulated in tumor tissues compared with normal tissues in breast, colon, liver, lung, pancreatic, and thyroid cancers. Secondly, we found that the gene signature, VAG, which is associated with apoptosis and composed of *VDAC1* and its interacting genes, is capable of predicting recurrence-free survival in breast, colon, and lung cancers. Finally, we confirmed that VAG is independent of standard clinical and pathological prognostic factors, which enhances the identification of cancer patients at greater risk for recurrence.

Among the gene list for VAG, *BCL2L1* and *CYCS* are among the top genes that have been heavily documented for their active roles in mitochondria-mediated apoptosis. *BCL2L1* encodes Bcl-2-like protein 1 in humans. This protein is located on the outer mitochondrial membrane and has been shown to regulate the opening of VDAC-1; thus, acting as an anti- or pro-apoptotic regulator. *BCL2L1* was found to play a functional role in colon [51], [52], prostate [53], [54], and pancreatic [55] cancers. *CYCS* encodes a small heme protein (cytochrome c) that functions as a central component of the electron transport chain in mitochondria. This protein is involved in the initiation of apoptosis. *CYCS* has been reported to be related to breast [56] and colon [57] cancers.

In contrast to many published prognostic gene signatures derived from whole genome screening, the VAG signature was developed based on one gene and pre-identified gene-gene interactions. Statistically-derived clinical signatures by whole genome screening are often highly accurate in the patient data sets from which they were identified, yet most of them have not been validated as useful clinical tools [58]. Moreover, a recent study suggests that random gene signatures have a high probability of being associated with survival outcome in cancer and most published signatures are not significantly more associated with outcome than random predictors [48]. In our study, the resampling test proved that the prognostic power of the VAG signature is significantly better than random gene signatures. More importantly, the genes discovered through our “bottom-up” approach have allowed the discovery of specific pathways relevant to human cancers and may yield new insights into disease pathology [59]. Our results identified that apoptosis associated genes are commonly differentially expressed between normal and tumor tissues in human carcinomas. The relationship between apoptosis and human cancers has been heavily documented in the past decade [60], [61], [62]. Thus, it is not surprising that the apoptosis associated gene signature is related to the aggressiveness of cancer across different tumor types. However, our results support the concept that mitochondria can serve as promising pharmacological targets in oncology [11]. More specifically, the emerging data on *VDAC1* associated genes will help to not only predict clinical outcome in diversified human cancers, but also identify specific drugs that can be used to obtain maximal cancer-killing efficacy [11].

## Conclusions

The expression level of *VDAC1* is uniformly up-regulated in tumor tissue compared with normal tissue in breast, colon, liver, lung, pancreatic, and thyroid cancers. The apoptosis associated molecular signature VAG, which is composed of *VDAC1* and some of its interacting genes, represents a promising prognostic biomarker in human cancers. When working cooperatively with standard clinical and pathological prognostic factors, VAG may enhance prediction accuracy in identifying patients at higher risk for recurrence. Future therapies aimed specifically at *VDAC1* associated genes may lead to novel agents in the treatment of cancer.

## Supporting Information

Figure S1 **Non-random inherent relationship in gene expression among** ***VDAC1*** **and its interacting genes.** We computed the gene regulatory network for VAG using GENIE3. Weighted adjacency matrix was computed for breast, colon, liver, lung, pancreatic, and thyroid cancers, respectively. We used the sum of weighted adjacency matrix to measure the inherent relationship in gene expression among *VDAC1* and its interacting genes. We also generated 1,000 random gene signatures with identical size as VAG. Weighted adjacency matrix was computed for each resampled gene set using GENIE3. The sum of weighted adjacency matrix of VAG is significantly larger than that of randomized signature.
(PDF)

Figure S2 **Kaplan-Meier curves for the patients in the training cohorts.** The expression of VAG predicts poor recurrence-free survival in breast, colon, and lung cancers. Red curves are for the VAG-positive patients while blue curves are for the VAG-negative patients. VAG-positive patients were defined as those having a risk score greater than the group median. *P*-values were calculated by log-rank tests for the differences in survival between the VAG-positive and -negative groups.
(PDF)

Figure S3 **Better prognostic power of VAG compared with other cancer-related genes.** *Z* denotes the Wald statistic. The black triangle stands for the sum of the *Z* values of VAG in the three validation cohorts. The grey area shows the distribution of the sum of the *Z* values for the 1,000 resampled gene signatures that are composed of the cancer-related genes. One-tailed *P*-value for the right tail of the sampling distribution was calculated.
(PDF)

Table S1 **Gene expression datasets used in this study.**
(PDF)

Table S2 **Interacting genes for** ***VDAC1*** **.**
(PDF)

Table S3 ***VDAC1*** **interacting genes that are differentially expressed between normal and tumor tissues.**
(PDF)

Table S4 **Cox proportional hazards regression of survival against** ***VDAC1*** **expression in breast, colon, and lung cancers.**
(PDF)

Table S5 **Cancer-related genes that don’t interact with** ***VDAC1*** **.**
(XLS)

Data S1 **The preprocessed training and validation datasets.**
(ZIP)
