# Supplementary figures and images for: Expression Profiling of Mitochondrial Voltage-Dependent Anion Channel-1 Associated Genes Predicts Recurrence-Free Survival in Human Carcinomas
Source: PLoS One. 2014 Oct 15;9(10):e110094. doi: 10.1371/journal.pone.0110094 (PMC4198298; doi:10.1371/journal.pone.0110094)

Breast

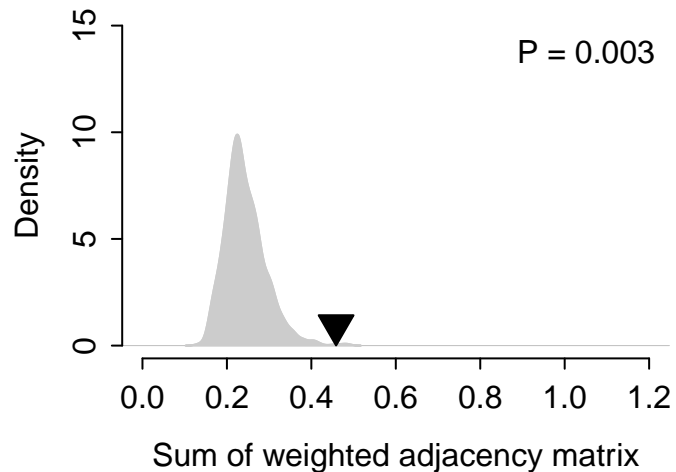

Colon

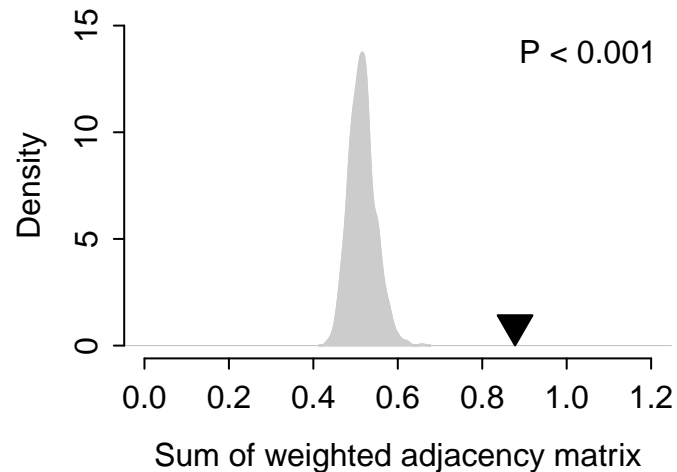

Liver

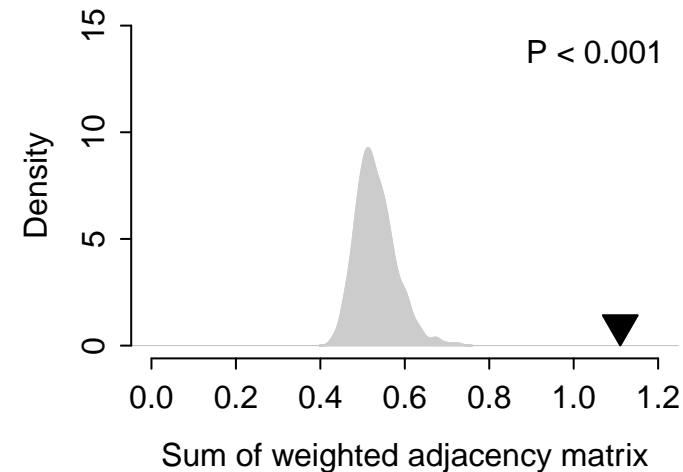

Lung

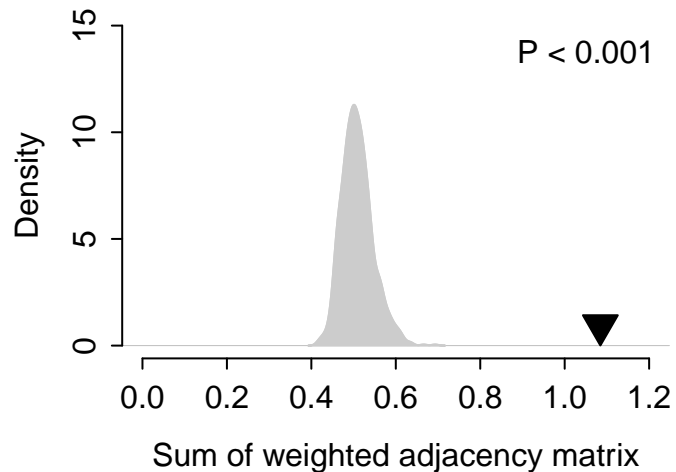

Pancreatic

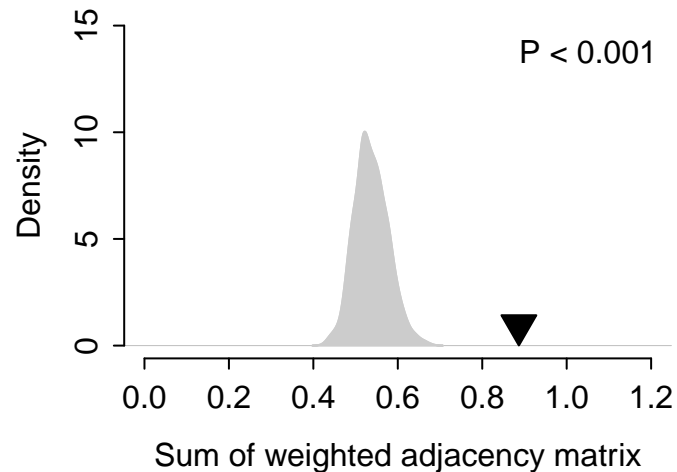

Thyroid

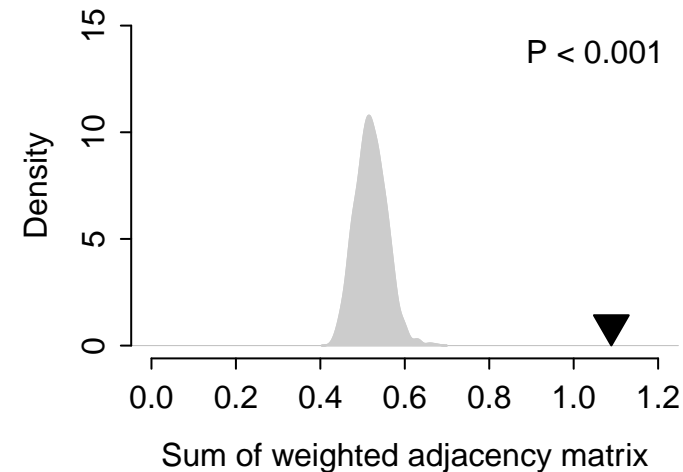

Supplement: Figure S1 — Non-random inherent relationship in gene expression among VDAC1 and its interacting genes. We computed the gene regulatory network for VAG using GENIE3. Weighted adjacency matrix was computed for breast, colon, liver, lung, pancreatic, and thyroid cancers, respectively. We used the sum of weighted adjacency matrix to measure the inherent relationship in gene expression among VDAC1 and its interacting genes. We also generated 1,000 random gene signatures with identical size as VAG. Weighted adjacency matrix was computed for each resampled gene set using GENIE3. The sum of weighted adjacency matrix of VAG is significantly larger than that of randomized signature. (PDF) [file pone.0110094.s001.pdf]

Breast

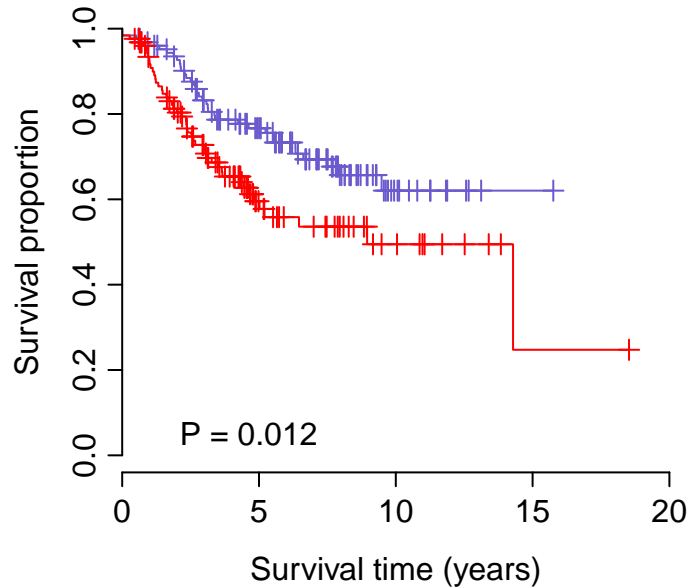

Colon

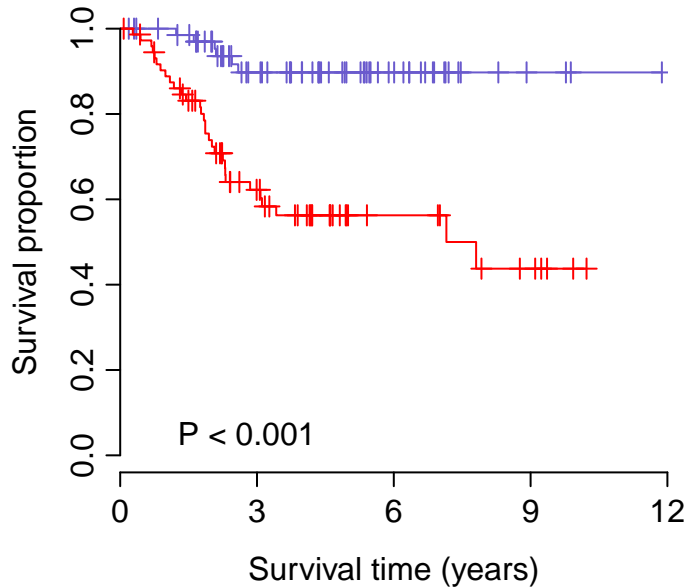

Lung

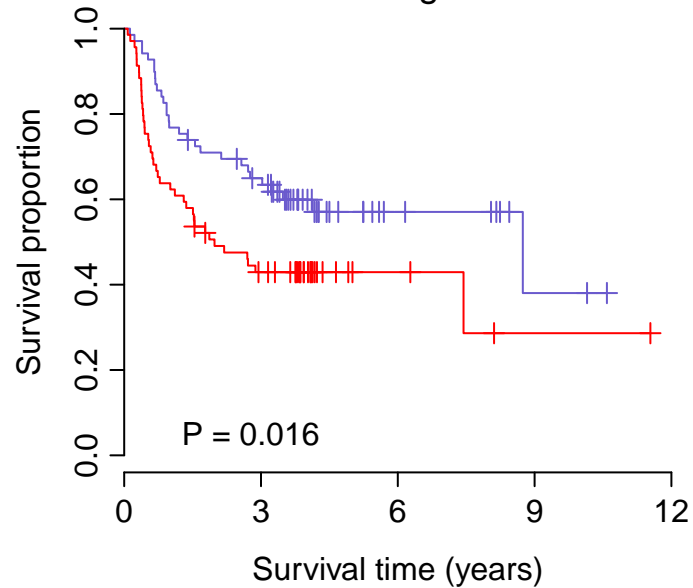

Supplement: Figure S2 — Kaplan-Meier curves for the patients in the training cohorts. The expression of VAG predicts poor recurrence-free survival in breast, colon, and lung cancers. Red curves are for the VAG-positive patients while blue curves are for the VAG-negative patients. VAG-positive patients were defined as those having a risk score greater than the group median. P-values were calculated by log-rank tests for the differences in survival between the VAG-positive and -negative groups. (PDF) [file pone.0110094.s002.pdf]

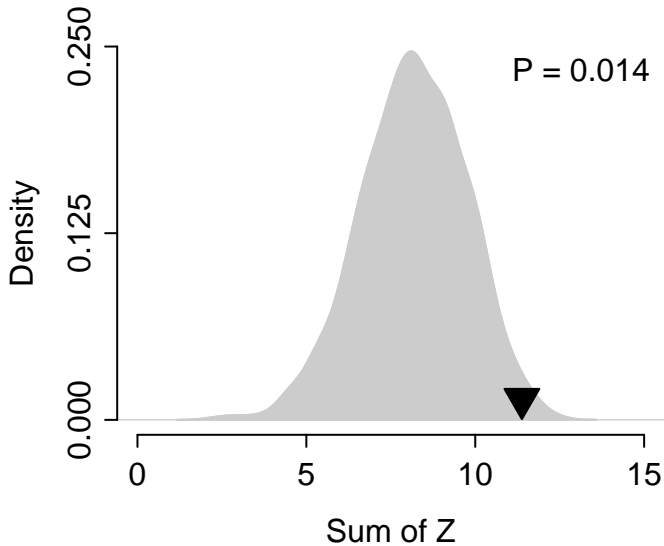

Supplement: Figure S3 — Better prognostic power of VAG compared with other cancer-related genes. Z denotes the Wald statistic. The black triangle stands for the sum of the Z values of VAG in the three validation cohorts. The grey area shows the distribution of the sum of the Z values for the 1,000 resampled gene signatures that are composed of the cancer-related genes. One-tailed P-value for the right tail of the sampling distribution was calculated. (PDF) [file pone.0110094.s003.pdf]
