# Supplementary material for: Expression Profiling of Mitochondrial Voltage-Dependent Anion Channel-1 Associated Genes Predicts Recurrence-Free Survival in Human Carcinomas
Source: PLoS One. 2014 Oct 15;9(10):e110094. doi: 10.1371/journal.pone.0110094 (PMC4198298; doi:10.1371/journal.pone.0110094)
Supplement: Table S1 — Gene expression datasets used in this study. (PDF) [file pone.0110094.s004.pdf]

Table S1. Gene expression datasets used in this study

	Cancer	GEO accession	Platform
Expression data of paired normal-tumor tissues	Breast	GSE15852	Affymetrix Human Genome U133A Array
	Colon	GSE23878	Affymetrix Human Genome U133 Plus 2.0 Array
	Liver	GSE14520	Affymetrix HT Human Genome U133A Array
	Lung	GSE18842	Affymetrix Human Genome U133 Plus 2.0 Array
	Pancreatic	GSE15471	Affymetrix Human Genome U133 Plus 2.0 Array
	Thyroid	GSE33630	Affymetrix Human Genome U133 Plus 2.0 Array
Training cohorts for recurrence-free survival analysis	Breast	GSE21653	Affymetrix Human Genome U133 Plus 2.0 Array
	Colon	GSE17536	Affymetrix Human Genome U133 Plus 2.0 Array
	Lung	GSE8894	Affymetrix Human Genome U133 Plus 2.0 Array
Testing cohorts for recurrence-free survival analysis	Breast	GSE25066	Affymetrix Human Genome U133A Array
	Colon	GSE39582	Affymetrix Human Genome U133 Plus 2.0 Array
	Lung	GSE31210	Affymetrix Human Genome U133 Plus 2.0 Array
