# Supplementary material for: Expression Profiling of Mitochondrial Voltage-Dependent Anion Channel-1 Associated Genes Predicts Recurrence-Free Survival in Human Carcinomas
Source: PLoS One. 2014 Oct 15;9(10):e110094. doi: 10.1371/journal.pone.0110094 (PMC4198298; doi:10.1371/journal.pone.0110094)
Supplement: Table S2 — Interacting genes for VDAC1 . (PDF) [file pone.0110094.s005.pdf]

Table S2. Interacting genes for *VDAC1*

Gene symbol	Interaction details	Gene symbol	Interaction details
A2M	MINT-4054731	*AARS2*	MINT-8079030
AASS	MINT-8079030	*ABCB7*	MINT-8079030
ACAA2	BioGRID	*ACAD9*	MINT-8079030
ACADVL	MINT-8079030	*ACAT2*	I2D: score=1 STRING: ENSP00000356015
ACOT9	MINT-8079030	*ACTB*	MINT-4054731 I2D: score=1 STRING: ENSP00000349960
ADCK3	MINT-8079030	*ADRB2*	BioGRID
AFG3L2	MINT-8079030	*AGK*	MINT-8079030
AIFM1	MINT-8079030	*AKAP1*	MINT-6543750
ALDH18A1	MINT-8079030	*ALDH1B1*	MINT-8079030
ALDH1L2	MINT-8079030	*ALDH2*	MINT-8079030
ALDH5A1	BioGRID	*ALPP*	MINT-4054731
AP2M1	MINT-8079030	*APOD*	MINT-4054731
ARL8B	MINT-4054731	*ASAH1*	MINT-4054731
ASB14	BioGRID	*ASB17*	BioGRID
ATAD3A	MINT-8079030	*ATAD3B*	MINT-8079030
ATF2	STRING: ENSP00000264110	*ATP5A1*	MINT-8079030 MINT-4054731 STRING: ENSP00000282050
ATP5B	MINT-8079030 MINT-4054731 STRING: ENSP00000262030	*ATP5C1*	MINT-8079030 STRING: ENSP00000349142
ATP5D	STRING: ENSP00000215375	*ATP5F1*	MINT-8079030 STRING: ENSP00000358737
ATP5H	MINT-8079030	*ATP5L*	MINT-8079030
ATP5O	STRING: ENSP00000290299	*ATP6V0D1*	MINT-4054731
ATP6V1A	MINT-4054731	*ATP6V1B2*	MINT-4054731 STRING: ENSP00000276390
AZU1	MINT-4054731	*BAG3*	BioGRID
BAK1	MINT-15123 MINT-15124 I2D: score=4 STRING: ENSP00000363591	*BAX*	I2D: score=5 STRING: ENSP00000293288
BCL2	STRING: ENSP00000329623	*BCL2L1*	I2D: score=3 STRING: ENSP00000302564
BCL2L11	I2D: score=3	*C10orf2*	MINT-8079030
C11orf68	BioGRID	*C1QBP*	MINT-8079030
C6orf203	MINT-8079030	*C9orf163*	I2D: score=1 STRING: ENSP00000346345
CANX	MINT-8374764	*CAPN6*	MINT-4054731
CASP4	I2D: score=1	*CAV1*	BioGRID

Gene symbol	Interaction details	Gene symbol	Interaction details
CBR1	I2D: score=1 STRING: ENSP00000290349	*CD4*	I2D: score=1
CDK2	BioGRID	*CECR5*	MINT-8079030
CHCHD1	MINT-8079030	*CKMT1A*	MINT-4054731 I2D: score=2
CKMT1B	MINT-4054731 I2D: score=2 STRING: ENSP00000300283	*CLN3*	I2D: score=1
CLPX	MINT-8079030	*COX4I1*	MINT-8079030
CPT2	MINT-8079030	*CSNK2B*	MINT-8253770 I2D: score=2
CTSG	MINT-4054731	*CYCS*	I2D: score=1 STRING: ENSP00000307786
CYP11A1	MINT-4054731	*CYP19A1*	MINT-4054731
DAP3	MINT-8079030	*DBT*	MINT-8079030
DCD	I2D: score=2 STRING: ENSP00000293371	*DDOST*	MINT-4054731
DDX28	MINT-8079030	*DENR*	STRING: ENSP00000280557
DHRS2	I2D: score=1	*DHX30*	MINT-8079030
DLD	MINT-8079030	*DLG4*	I2D: score=1
DLST	MINT-4054731	*DNAJA3*	MINT-8079030
DYNLT1	I2D: score=3 STRING: ENSP00000356056	*DYNLT3*	I2D: score=3 STRING: ENSP00000367841
ECH1	MINT-8079030	*ECI1*	MINT-8079030
EIF6	I2D: score=1	*ENDOG*	STRING: ENSP00000361725
ENV	BioGRID	*ERAL1*	MINT-8079030
ERLIN2	MINT-8079030	*ETFA*	MINT-8079030
FARS2	MINT-8079030	*FASTKD1*	MINT-8079030
FASTKD2	MINT-8079030	*FLAD1*	MINT-8079030
FLOT2	BioGRID	*FMNL1*	BioGRID
FSCN1	I2D: score=1 STRING: ENSP00000371798	*GABARAPL2*	I2D: score=2
GADD45GIP1	MINT-8079030	*GANAB*	I2D: score=1 STRING: ENSP00000340466
GAPDH	MINT-4054731	*GBA*	MINT-4054731
GLB1	MINT-4054731	*GLDC*	MINT-8079030
GLUD1	MINT-8079030	*GOT2*	I2D: score=1 STRING: ENSP00000245206
GRIN2B	I2D: score=1	*GRPEL1*	MINT-8079030
GSN	MINT-4792996 I2D: score=4 STRING: ENSP00000362924	*GSTK1*	I2D: score=1
GTPBP10	MINT-8079030	*GUSB*	MINT-4054731
HADHA	MINT-8079030	*HARS2*	MINT-8079030
HAUS3	BioGRID	*HDAC5*	I2D: score=1 STRING: ENSP00000225983
HK1	EBI-354158,EBI-713162 MINT-8079030 I2D: score=1	*HK2*	Manually added

Gene symbol	Interaction details	Gene symbol	Interaction details
HLA-B	I2D: score=1	*HNRNPDL*	MINT-8079030
HSD17B10	MINT-8079030	*HSPA1A*	MINT-8079030
HSPA1B	MINT-8079030	*HSPA5*	MINT-4054731
HSPA9	MINT-8079030 I2D: score=2	*HSPD1*	MINT-8079030 MINT-4054731 STRING: ENSP00000340019
HTT	BioGRID	*ICT1*	MINT-8079030 I2D: score=1 STRING: ENSP00000301585
IGF2BP2	MINT-8079030	*IKBKB*	STRING: ENSP00000339151
IKBKE	I2D: score=1	*ISCA1*	MINT-8079030
KARS	MINT-8079030	*KCNMA1*	I2D: score=1
KIAA0391	MINT-8079030	*KIF5B*	I2D: score=2 STRING: ENSP00000307078
LETM1	MINT-8079030	*LONP1*	MINT-8079030
LRPPRC	MINT-8079030	*MALSU1*	MINT-8079030
MAOA	MINT-4054731	*MAPK1*	STRING: ENSP00000215832
MAPK3	I2D: score=1 STRING: ENSP00000263025	*MCAT*	MINT-8079030
MCC	I2D: score=2	*MCCC2*	MINT-8079030
MCL1	I2D: score=3 STRING: ENSP00000358022	*MCU*	MINT-8079030
MDC1	BioGRID	*MDM2*	BioGRID
ME2	MINT-8079030	*METTL17*	MINT-8079030
MGAT4B	BioGRID	*MMAB*	MINT-8079030
MMP2	Manually added	*MMP9*	Manually added
MOV10	MINT-8079030	*MPO*	MINT-4054731
MRM1	MINT-8079030	*MRPL1*	MINT-8079030
MRPL10	MINT-8079030	*MRPL11*	MINT-8079030
MRPL12	MINT-8079030	*MRPL13*	MINT-8079030
MRPL14	MINT-8079030	*MRPL15*	MINT-8079030
MRPL16	MINT-8079030	*MRPL17*	MINT-8079030
MRPL18	MINT-8079030	*MRPL19*	MINT-8079030
MRPL2	MINT-8079030	*MRPL20*	MINT-8079030
MRPL21	MINT-8079030	*MRPL22*	MINT-8079030
MRPL24	MINT-8079030	*MRPL27*	MINT-8079030
MRPL28	MINT-8079030	*MRPL3*	MINT-8079030
MRPL32	MINT-8079030	*MRPL37*	MINT-8079030
MRPL39	MINT-8079030	*MRPL4*	MINT-8079030
MRPL40	MINT-8079030	*MRPL41*	MINT-8079030

Gene symbol	Interaction details	Gene symbol	Interaction details
MRPL43	MINT-8079030	*MRPL44*	MINT-8079030
MRPL46	MINT-8079030	*MRPL47*	MINT-8079030
MRPL48	MINT-8079030	*MRPL49*	MINT-8079030
MRPL50	MINT-8079030	*MRPL51*	MINT-8079030
MRPL54	MINT-8079030	*MRPL55*	MINT-8079030
MRPL9	MINT-8079030	*MRPS10*	MINT-8079030
MRPS11	MINT-8079030	*MRPS14*	MINT-8079030
MRPS15	MINT-8079030	*MRPS16*	MINT-8079030
MRPS17	MINT-8079030	*MRPS18A*	MINT-8079030
MRPS18B	MINT-8079030	*MRPS2*	MINT-8079030
MRPS21	MINT-8079030	*MRPS22*	MINT-8079030
MRPS23	MINT-8079030	*MRPS24*	MINT-8079030
MRPS25	MINT-8079030	*MRPS26*	MINT-8079030
MRPS27	MINT-8079030	*MRPS28*	MINT-8079030
MRPS30	MINT-8079030	*MRPS34*	MINT-8079030
MRPS35	MINT-8079030	*MRPS5*	MINT-8079030
MRPS6	MINT-8079030	*MRPS7*	MINT-8079030
MRPS9	MINT-8079030	*MRRF*	MINT-8079030
MT-CO2	MINT-8079030	*MTERF*	MINT-8079030
MTERFD1	MINT-8079030	*MTG1*	MINT-8079030
MTPAP	MINT-8079030	*MUT*	MINT-8079030
NDUFA9	MINT-8079030	*NDUFAF3*	MINT-8079030
NDUFS1	MINT-8079030	*NDUFS2*	MINT-8079030
NDUFS3	MINT-8079030	*NDUFS8*	MINT-8079030
NDUFV1	MINT-8079030	*NGRN*	MINT-8079030
NME4	MINT-8079030	*NOA1*	MINT-8079030
NSUN4	MINT-8079030	*NUDC*	I2D: score=1 STRING: ENSP00000319664
OAT	MINT-8079030	*PANK2*	I2D: score=2 STRING: ENSP00000313377
PARK2	BioGRID	*PARS2*	MINT-8079030
PCCB	MINT-8079030	*PDK3*	MINT-8079030
PGAM5	MINT-8079030	*PHB*	MINT-8079030 STRING: ENSP00000300408
PHB2	MINT-8079030 STRING: ENSP00000382362	*PMPCA*	MINT-8079030
PMPCB	MINT-8079030	*PNPT1*	MINT-8079030
POLDIP2	MINT-8079030	*POLRMT*	MINT-8079030
PPID	STRING: ENSP00000303754	*PPIF*	I2D: score=2 STRING: ENSP00000225174

Gene symbol	Interaction details	Gene symbol	Interaction details
PRDX4	MINT-8079030	*PRDX5*	MINT-8079030
PRKCE	I2D: score=3 STRING: ENSP00000306124	*PRTN3*	MINT-4054731
PTCD1	MINT-8079030	*PTCD3*	MINT-8079030
PUS1	MINT-8079030	*PUSL1*	MINT-8079030
PYCR2	MINT-8079030	*RAF1*	I2D: score=2 STRING: ENSP00000251849
RARS2	MINT-8079030	*RBFA*	MINT-8079030
RIPK2	I2D: score=1	*RNGTT*	STRING: ENSP00000358497
RNMTL1	MINT-8079030	*RPUSD3*	MINT-8079030
RPUSD4	MINT-8079030	*SARS2*	MINT-8079030
SCARB2	MINT-4054731	*SF1*	BioGRID
SF3A1	I2D: score=1 STRING: ENSP00000215793	*SFXN1*	MINT-8079030
SFXN3	BioGRID	*SHMT2*	MINT-8079030
SIRT7	STRING: ENSP00000329466	*SLC25A1*	MINT-8079030
SLC25A10	MINT-8079030	*SLC25A11*	MINT-8079030
SLC25A12	MINT-8079030	*SLC25A13*	MINT-8079030
SLC25A18	MINT-8079030	*SLC25A3*	MINT-8079030
SLC25A31	STRING: ENSP00000281154	*SLC25A4*	MINT-8079030 STRING: ENSP00000281456
SLC25A5	MINT-8079030 MINT-4054731 STRING: ENSP00000360671	*SLC25A6*	MINT-4054731 STRING: ENSP00000370808
SLC2A4	I2D: score=1	*SLIRP*	MINT-8079030
SNCA	I2D: score=2 STRING: ENSP00000338345	*SSBP1*	MINT-8079030
STOML2	MINT-8079030	*STS*	MINT-4054731
SUCLA2	MINT-8079030	*SUGP1*	BioGRID
SUMO4	I2D: score=1 STRING: ENSP00000318635	*SUPV3L1*	MINT-8079030
TARS2	MINT-8079030	*TEFM*	MINT-8079030
TFAM	MINT-8079030	*TFB1M*	MINT-8079030
TIAL1	BioGRID	*TIMM21*	MINT-8079030
TMX1	MINT-8374764	*TOMM20*	I2D: score=3 STRING: ENSP00000355566
TOP1	MINT-8079030	*TPM3*	I2D: score=1
TPP1	MINT-4054731	*TRAF2*	STRING: ENSP00000247668
TRAF6	I2D: score=2	*TRMT10C*	MINT-8079030
TRUB2	MINT-8079030	*TTN*	I2D: score=1
TUBA1B	BioGRID	*TUBA4A*	I2D: score=2 STRING: ENSP00000248437
UBA52	BioGRID	*UBAP2*	BioGRID
UBC	I2D: score=1 STRING: ENSP00000344818	*UFM1*	BioGRID

Gene symbol	Interaction details	Gene symbol	Interaction details
UQCR10	STRING: ENSP00000332887	*UQCRC1*	MINT-8079030
UQCRC2	MINT-8079030	*UQCRFS1*	MINT-8079030
VAPA	BioGRID	*VARS2*	MINT-8079030
VCAM1	BioGRID	*VDAC2*	MINT-8374764
VDAC3	MINT-8079030	*WBSCR16*	MINT-8079030
YARS2	MINT-8079030	*YME1L1*	MINT-8079030
YWHAB	I2D: score=1	*ZMPSTE24*	BioGRID
