# Supplementary material for: Expression Profiling of Mitochondrial Voltage-Dependent Anion Channel-1 Associated Genes Predicts Recurrence-Free Survival in Human Carcinomas
Source: PLoS One. 2014 Oct 15;9(10):e110094. doi: 10.1371/journal.pone.0110094 (PMC4198298; doi:10.1371/journal.pone.0110094)
Supplement: Table S3 — VDAC1 interacting genes that are differentially expressed between normal and tumor tissues. (PDF) [file pone.0110094.s006.pdf]

Table S3. *VDAC1* interacting genes that are differentially expressed between normal and tumor tissues

	Breast		Colon		Liver		Lung		Pancreatic		Thyroid	
Gene symbol	*FC*	Adjusted *P*	*FC*	Adjusted *P*	*FC*	Adjusted *P*	*FC*	Adjusted *P*	*FC*	Adjusted *P*	*FC*	Adjusted *P*
VDAC1	1.25	4.9E-03	1.32	3.8E-02	1.64	1.7E-25	1.67	2.0E-10	1.91	1.5E-03	1.14	1.0E-02
ACADVL	-	-	0.51	6.9E-04	0.42	1.5E-47	0.64	5.9E-08	0.70	6.9E-05	1.27	7.1E-05
AGK	-	-	1.58	4.4E-03	1.88	3.6E-35	1.85	2.9E-11	1.21	2.0E-02	1.17	3.7E-03
AP2M1	-	-	0.90	4.7E-01	1.49	1.3E-15	1.82	2.0E-08	2.03	5.6E-06	1.15	8.6E-04
ATP6V1A	1.10	1.8E-02	0.87	2.4E-01	1.39	1.9E-18	1.74	8.9E-05	1.34	1.6E-02	0.81	1.5E-01
BCL2L1	-	-	1.86	4.7E-04	1.19	1.3E-06	0.72	2.9E-04	1.55	2.7E-05	1.97	2.4E-10
CDK2	1.03	5.0E-01	1.81	7.0E-03	1.74	4.3E-26	1.70	1.9E-11	1.36	2.7E-03	1.08	2.9E-01
COX4I1	1.04	5.9E-01	0.63	1.2E-02	0.86	7.8E-05	0.97	3.7E-01	0.88	3.6E-03	0.67	3.6E-05
CYCS	-	-	0.37	4.7E-04	-	-	0.39	6.9E-08	0.72	9.7E-03	0.43	3.5E-10
DAP3	-	-	1.44	3.3E-03	2.76	8.3E-75	1.70	2.6E-15	1.13	7.1E-02	1.29	2.4E-04
DBT	1.43	4.1E-05	0.88	4.0E-01	0.62	2.6E-24	0.61	7.5E-09	0.62	8.2E-05	0.51	6.3E-08
DENR	2.40	1.2E-05	1.65	5.2E-03	1.92	1.3E-43	2.02	1.6E-20	1.20	1.7E-03	0.81	4.1E-02
DHX30	-	-	2.03	1.6E-02	1.25	6.5E-21	1.12	8.0E-02	1.25	4.5E-04	1.13	3.7E-02
ECI1	-	-	0.70	1.9E-02	0.66	7.2E-14	1.20	6.4E-03	0.80	1.7E-02	0.69	4.3E-05
EIF6	-	-	2.12	6.6E-04	1.60	1.4E-33	2.08	7.8E-15	1.52	9.3E-04	1.03	5.0E-01
FLAD1	1.00	5.5E-01	1.72	5.9E-04	2.13	4.6E-45	2.61	7.6E-14	1.30	3.8E-03	1.12	1.7E-02
GAPDH	1.58	1.6E-05	1.18	1.8E-02	1.71	7.2E-28	2.82	3.0E-22	1.84	2.4E-05	1.28	1.0E-07
GSN	0.39	8.0E-06	0.35	9.7E-04	0.58	9.6E-11	0.64	5.6E-05	2.24	4.6E-06	1.04	8.1E-01
GSTK1	-	-	0.78	4.9E-02	0.80	1.4E-06	0.56	1.5E-10	1.11	2.9E-01	0.70	3.1E-10
HADHA	-	-	0.72	4.7E-04	0.76	6.5E-08	0.87	2.4E-03	0.75	1.2E-02	0.78	3.1E-05
HAUS3	-	-	1.44	4.9E-02	1.84	1.7E-26	1.30	1.4E-04	1.30	1.8E-02	0.97	5.8E-01
IGF2BP2	-	-	1.68	1.3E-02	5.25	4.5E-36	3.52	2.1E-07	2.35	1.9E-05	4.56	1.8E-12
KIAA0391	1.00	7.0E-01	1.09	7.1E-01	1.21	6.5E-06	1.51	1.0E-07	1.18	1.5E-02	1.14	1.8E-02
KIF5B	1.03	8.3E-01	1.48	2.8E-02	1.71	1.1E-28	1.61	1.1E-04	1.30	6.7E-03	1.14	7.4E-04
LONP1	-	-	2.02	1.2E-04	1.16	1.4E-04	1.99	1.9E-12	1.39	4.8E-04	1.14	7.6E-02
MAPK1	-	-	0.77	9.6E-02	2.65	5.7E-52	2.03	1.7E-10	1.46	8.9E-03	2.02	2.0E-06
MCAT	-	-	1.70	3.3E-03	1.11	1.5E-02	1.60	1.3E-05	1.21	2.1E-02	0.86	5.5E-03
MCL1	1.06	3.8E-01	0.63	1.1E-02	0.60	1.2E-12	0.48	7.5E-12	1.41	5.5E-04	0.62	2.8E-02
MDC1	-	-	1.79	1.6E-03	1.74	2.0E-28	1.36	1.4E-03	1.42	9.5E-05	0.55	3.1E-10
MRPL3	1.84	8.7E-05	1.65	7.8E-04	1.46	1.2E-22	2.93	1.2E-12	1.02	8.9E-01	0.89	2.2E-02
MRPL9	-	-	1.64	2.1E-04	2.59	1.1E-71	1.59	1.5E-11	1.09	1.8E-01	1.12	3.6E-03
MRPS10	-	-	1.42	1.7E-02	1.50	7.6E-30	2.05	3.6E-13	1.16	9.2E-03	0.67	2.3E-06

	Breast		Colon		Liver		Lung		Pancreatic		Thyroid	
Gene symbol	*FC*	Adjusted *P*	*FC*	Adjusted *P*	*FC*	Adjusted *P*	*FC*	Adjusted *P*	*FC*	Adjusted *P*	*FC*	Adjusted *P*
MRPS17	-	-	2.87	4.7E-04	2.28	6.3E-49	2.77	4.5E-15	1.32	5.4E-03	0.87	3.2E-03
MTERFD1	-	-	1.98	9.4E-04	2.02	9.8E-39	1.78	1.5E-08	1.47	1.4E-04	0.92	1.2E-01
MTPAP	-	-	2.15	3.5E-03	1.45	1.2E-17	2.56	7.5E-07	1.15	1.5E-02	0.80	5.5E-11
MUT	-	-	0.61	3.1E-03	0.41	6.5E-43	0.67	6.3E-06	0.77	5.6E-04	0.61	6.0E-10
PANK2	-	-	1.33	6.1E-02	1.27	4.5E-11	1.14	3.3E-02	1.48	3.6E-05	1.25	5.1E-08
PPID	-	-	0.19	5.7E-06	0.92	4.4E-02	0.31	1.4E-15	0.32	2.5E-06	0.53	8.8E-10
RNGTT	1.04	1.9E-02	1.14	4.4E-01	1.39	4.7E-19	2.25	1.0E-12	1.23	4.5E-02	0.53	2.0E-04
TFB1M	-	-	1.59	2.4E-02	0.86	2.8E-04	1.63	9.0E-07	1.37	4.5E-04	1.19	4.8E-02
TIAL1	1.13	4.9E-03	1.31	6.0E-03	1.44	1.8E-21	1.63	6.6E-11	1.45	2.0E-05	0.74	2.1E-06
TMX1	1.57	1.6E-03	0.61	1.4E-01	1.75	1.1E-15	1.85	1.2E-07	1.43	4.9E-03	0.51	4.6E-06
TOMM20	3.49	4.6E-06	1.37	3.3E-02	2.16	3.6E-42	1.56	2.1E-09	0.92	3.0E-01	0.69	3.8E-08
TUBA4A	1.25	6.0E-02	1.65	4.7E-04	1.87	3.2E-18	2.07	1.2E-10	2.39	1.8E-05	1.98	2.4E-07
YWHAB	2.14	4.9E-03	1.60	4.7E-04	1.97	4.0E-40	0.87	6.1E-04	1.87	2.7E-05	1.17	1.5E-06

Note - *FC* is the abbreviation of fold change; Paired t-tests were used to measure the difference in gene expression between normal and tumor tissues; *P*-values were adjusted by Benjamini & Hochberg correction; "-" means that the gene expression data are not available.
