# Supplementary material for: Expression Profiling of Mitochondrial Voltage-Dependent Anion Channel-1 Associated Genes Predicts Recurrence-Free Survival in Human Carcinomas
Source: PLoS One. 2014 Oct 15;9(10):e110094. doi: 10.1371/journal.pone.0110094 (PMC4198298; doi:10.1371/journal.pone.0110094)
Supplement: Table S4 — Cox proportional hazards regression of survival against VDAC1 expression in breast, colon, and lung cancers. (PDF) [file pone.0110094.s007.pdf]

Table S4. Cox proportional hazards regression of survival against *VDAC1* expression in breast, colon, and lung cancers

	Training cohort			Validation cohort		
Cancer	Hazard ratio	95% confidence interval	*P*-value	Hazard ratio	95% confidence interval	*P*-value
Breast	1.33	(0.86, 2.06)	0.195	1.12	(0.77, 1.62)	0.564
Colon	1.43	(0.89, 2.30)	0.139	1.48	(0.90, 2.46)	0.125
Lung	0.96	(0.50, 1.85)	0.908	0.92	(0.69, 1.24)	0.584

Note - Patients were divided into high- and low-risk groups with the median of *VDAC1* expression level as the threshold value.
